# Fc Engineering for Developing Therapeutic Bispecific Antibodies and Novel Scaffolds

**DOI:** 10.3389/fimmu.2017.00038

**Published:** 2017-01-26

**Authors:** Hongyan Liu, Abhishek Saxena, Sachdev S. Sidhu, Donghui Wu

**Affiliations:** ^1^Laboratory of Antibody Engineering, Shanghai Institute for Advanced Immunochemical Studies, ShanghaiTech University, Shanghai, China; ^2^Banting and Best Department of Medical Research, Terrence Donnelly Center for Cellular and Biomolecular Research, University of Toronto, Toronto, ON, Canada; ^3^Department of Molecular Genetics, Terrence Donnelly Center for Cellular and Biomolecular Research, University of Toronto, Toronto, ON, Canada

**Keywords:** mAbs, Fc region, FcRn, bispecific, monovalent, heterodimer, monomeric Fc, Fc antigen-binding

## Abstract

Therapeutic monoclonal antibodies have become molecules of choice to treat autoimmune disorders, inflammatory diseases, and cancer. Moreover, bispecific/multispecific antibodies that target more than one antigen or epitope on a target cell or recruit effector cells (T cell, natural killer cell, or macrophage cell) toward target cells have shown great potential to maximize the benefits of antibody therapy. In the past decade, many novel concepts to generate bispecific and multispecific antibodies have evolved successfully into a range of formats from full bispecific immunoglobulin gammas to antibody fragments. Impressively, antibody fragments such as bispecific T-cell engager, bispecific killer cell engager, trispecific killer cell engager, tandem diabody, and dual-affinity-retargeting are showing exciting results in terms of recruiting and activating self-immune effector cells to target and lyse tumor cells. Promisingly, crystallizable fragment (Fc) antigen-binding fragment and monomeric antibody or half antibody may be particularly advantageous to target solid tumors owing to their small size and thus good tissue penetration potential while, on the other hand, keeping Fc-related effector functions such as antibody-dependent cellular cytotoxicity, complement-dependent cytotoxicity, antibody-dependent cell-mediated phagocytosis, and extended serum half-life *via* interaction with neonatal Fc receptor. This review, therefore, focuses on the progress of Fc engineering in generating bispecific molecules and on the use of small antibody fragment as scaffolds for therapeutic development.

## Introduction

Since approval of the first therapeutic monoclonal antibody (mAb) muromonab-CD3 by the United States Food and Drug Administration for treatment of organ transplant-associated acute rejections in 1992, a total of 62 mAbs have been approved by the USFDA for clinical use as of May 2016 (1, 2). Therefore, the USFDA has approved an average of two to three mAbs each year over the last 25 years. Surprisingly, in 2015, a total of 10 mAbs were approved (2). Clearly, the demand for antibody molecules and global sales have been rising rapidly.

Most therapeutic mAbs are complete immunoglobulin gamma (IgG) molecules which consist of two heavy and two light chains that fold into a complex quaternary Y-shaped structure (1). The two arms of the Y-shaped molecule form the antigen-binding domains called antigen-binding fragment (Fab) regions, and the stalk forms the crystallizable fragment (Fc) region. Native IgG molecules can be digested by papain protease into separate F(ab)_2_ dimers and Fc domains (3). Fab arms are responsible for antigen binding and have been extensively engineered for developing highly specific and synthetic antibodies against numerous targets (4). The Fc region bears recognition motifs for binding innate immune receptors [Fcγ receptors (FcγRs), C1q, and neonatal Fc receptor (FcRn)] on an effector cell and thus is responsible for mediating immune effector functions and *in vivo* IgG stability (5–11). This part has been a prime molecular engineering target for either enhancing or inhibiting the immune response including antibody-dependent cellular cytotoxicity (ADCC), complement-dependent cytotoxicity (CDC), and antibody-dependent cell-mediated phagocytosis (ADCP) (9, 12–14). Besides the Fab domain, antigen-binding character has also been engineered into the Fc, CH2, and CH3 domains (15, 16). Such novel fragments have demonstrated therapeutic-like profiles in early studies and remain attractive ventures.

Antibody molecules can be made more efficient by engineering additional specificities so that multiple antigens or epitopes present on a cell can be targeted (17, 18). Extensive academic and industrial research in the past decade focused on developing bispecific Abs and Igs (bsAbs and bsIgs) and multispecific antibodies (e.g., TriMabs) (17–20).

**Figure 1:**
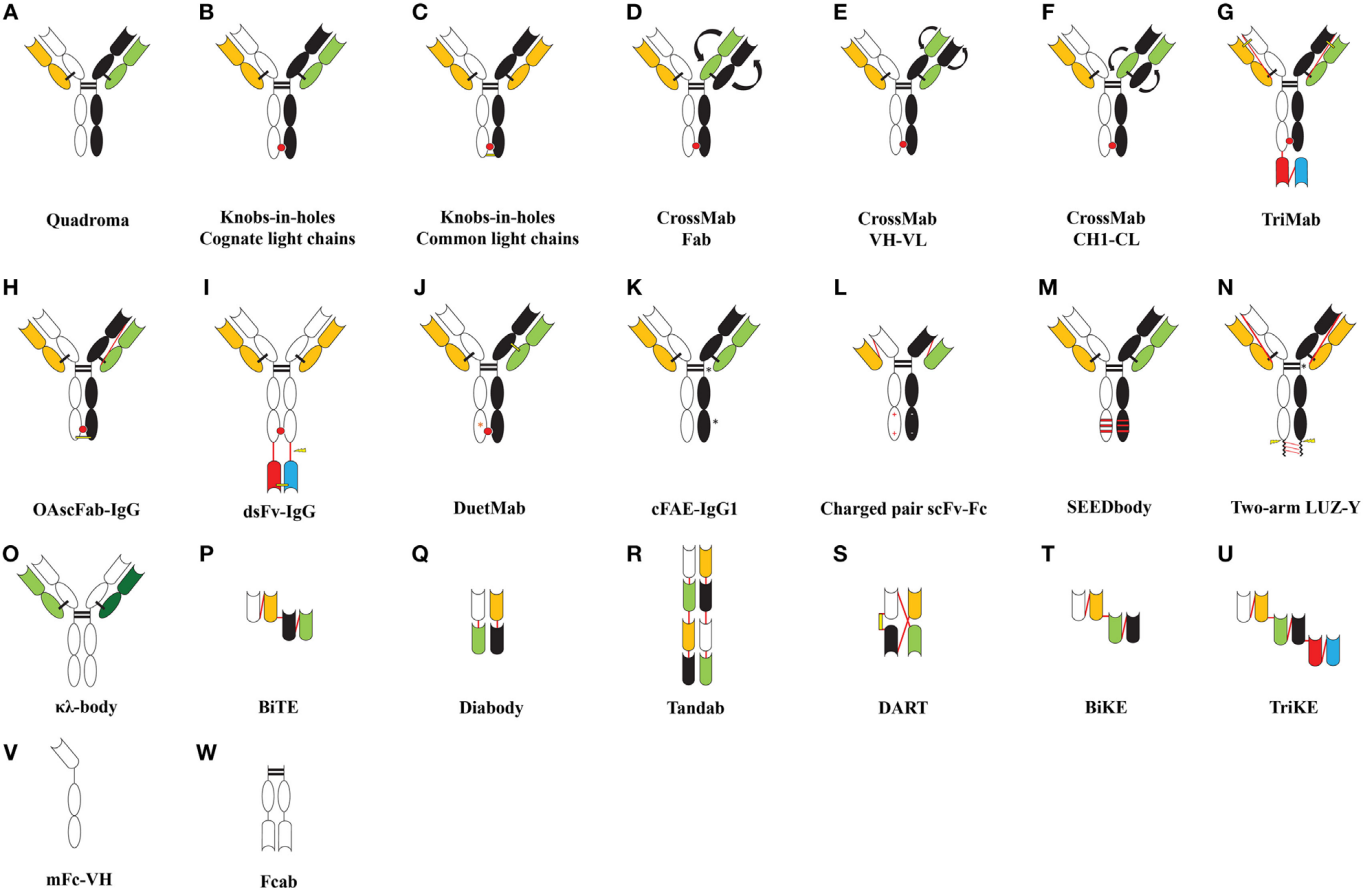
**Formats of bispecific antibodies and scaffolds described in the literature**. **(A)** Bispecific quadroma generated by somatic fusion of two hybridomas, **(B–J)** bispecific formats developed by using knobs-in-holes (KiH) Fc heterodimerization strategy, **(K)** bispecific IgG1 developed by controlled Fab-arm exchange, **(L)** bispecific Fc-fusion constructs developed by electrostatic optimization, **(M–O)** bispecific formats developed by strand exchange, insertion of cleavage motif and expressing two light chains with single heavy chain, and **(P–W)** novel bispecifc or multi-specific scaffolds. Abbreviations: OAscFab-IgG, one-arm single-chain Fab-immunoglobulin gamma (IgG); dsFv-IgG, disulfide stabilized Fv-IgG; cFAE-IgG1, controlled Fab-arm exchanged-IgG1; scFv-Fc, charged pair single-chain Fv-Fc fusion; SEEDbody, strand-exchange engineered domain body; LUZ-Y, two-arm leucine zipper heterodimeric monoclonal antibodies; (κλ-body, kappa lambda body; BiTEs, bispecific T-cell engagers; BiKEs/TriKEs, bispecific/trispecific killer cell engagers; DART, dual-affinity retargeting molecules; mFc, monomeric Fc; Fcab, Fc antigen binding. Color codes are as follows: heterodimeric CH domains are shown in black and white; cognate light chains are shown in green and orange; the antigen-binding domains [VH (black/white), VL (green/orange), and VH–VL (paired white–orange/black–green)] are shown as indented bubble; additional specificity in trispecific molecules is shown by red/blue scFv or VH–VL pair **(G,I,U)**; light chains of κλ-body **(O)** are shown in light and dark green; red bulge at CH3–CH3 interface depicts knobs-in-holes motif; curved arrows indicate cross-matched domains; interchain linkers are shown as red lines; interchain disulphide bonds are shown as black lines; engineered disulfide bonds are shown as yellow lines; red stripes depict exchanged sequences; black asterisks indicate site of mutations favoring heterodimerization; orange asterisks indicate protein A ablation mutation **(J)**. +/− represents charged pair mutations; lightning bolt indicates cleavable peptide linkers.

Initially, bsAbs were generated by a quadroma technology, which required the somatic fusion of two hybridomas harboring different specificities (21, 22). This led to the foundation of bispecific antibody production for simultaneous targeting of two different antigens or epitopes on a cell (Figure 1A). However, these molecules suffered from low production yields, heterogeneity, and human anti-mouse antibody (HAMA) response and therefore a decreased efficacy in patients (23). Nevertheless, one such bsAb EpCAM × CD3 (triomab; catumaxomab) was approved by the European Medicines Agency in 2009 for the treatment of patients with epithelial cancer-associated malignant ascites (24). Two additional bsAbs produced using the quadroma method, HER2 × CD3 (triomab; ertumaxomab) and CD20 × CD3 (triomab; FBTA05), are being evaluated in clinical trials for cancer treatment (25, 26).

Later, bsAb construction primarily relied on Fc heterodimerization by creating “knobs-in-holes” (KiH) mutations in the CH3 domain, which is prerequisite to assemble two half antibodies (common Fc heterodimer and unique VH–CH and VL–CL domains) (27–29). However, a major bottleneck in this strategy has been the incorrect pairing of light–heavy chains. Consequently, newer strategies based on KiH and other platforms have been developed to circumvent faulty heavy and light chain pairing.

Small antibody fragments like nanobodies from llama and camel immune systems (30–33), single human domain antibodies (34–37), and single-chain variable fragments (scFvs) (38, 39) can be used to impart bispecificity or multispecificity to antibody molecules (18). Moreover, small non-immunoglobulin fragments like monoclonal lamprey antibodies (lambodies) (40), affibodies (41), and DNA/RNA aptamers (42, 43) can be fused with the antibody Fc fragment (homodimerization or heterodimerization) to have antibody-like properties such as Fc-associated effector functions (ADCC, CDC, and ADCP), extended pharmacokinetics and bispecificity (44, 45).

In this review, we describe recent advances in the therapeutic potential of bispecific molecules and small Ab fragments as novel scaffolds. We summarize the key breakthroughs achieved by employing and optimizing various strategies. Representative design, expression, purification, and purity of final bispecific molecules are summarized (see Table 1). Some bispecific antibodies and antibody fragments currently under clinical evaluation are listed (see Table 2), and graphical models of bispecific molecules under development are presented (see Figure 1).

**Table 1:** **Strategies to promote bispecificity by heterodimer formation**.

Strategy/format	Mutation	Target	Bispecificity analysis/yield (%)	Protein expression/purification/yield (g/L)	Remarks	Reference
Quadroma	NA	EpCAM × CD3, HER3 × CD3, CD20 × CD3	Ion-exchange chromatography and SEC/12.5%	Hybridoma/protein A	Associated human anti-mouse antibody response	(26, 46)
Knobs-in-holes	T366W/T366Y (knobs) and T366S/L368A/T394W/F405A/Y407V(T) (holes)	CD3 × CD4, c-MPL × HER3, VEGF-A × Ang2, CD20 × hL243γ1, EGFR × IGF1R, HER3 × cMET, CD3 × EpCAM, CD3 × HER2, EGFR × HER2, CD4 × CD70, MET × EGFR	Electroblotting, SLD, SEC, MS-TOF (73–100%)	HEK293, *Escherichia coli*, and cell-free expression system/protein A, DEAE, and Mab Select Sure/0.004–1.0 g/L	Faulty light and heavy chain pairing	(19, 27–29, 47–53)
Biochemical optimization	S364H/F405A (CH3A) andY349T/T394F (CH3B)	CD16 × HER2 and CD3 × HER2	HPLC/SEC (89%)	HEK293F/protein A, nickel affinity chromatography		(54)
Biochemical optimization	L368E/K409R (CH3), R221E/R228E (IgG1-hinge), and R223E/R225E/R228E (IgG2-hinge)	CD3 × CD20, EGFR × ErbB2	Ion-exchange chromatography and LCMS (65–100%)	HEK293/protein A/*in vitro* cell-free assembly	Cognate chain pairing	(55)
Biochemical optimization	P228S (IgG1-hinge) and F405L/K409R (CH3)	CD20 × EGFR	ESI-MS (95.7%)	HEK293/protein A/SEC/473.4 g/L	High yield and efficiency	(56)
Biochemical optimization	H435R and Y436F (IgG1-CH3)	CD20 × CD3	SEC (100%)	Stable CHO-K1/protein A/0.2–0.3 g/L	Cognate chain pairing	(57, 58)
Biochemical optimization	S354C (CH3A), Y394C (CH3B), F126C (CH1), S121C (LC), C44 (VH), and C100 (VL)	EGFR × IGF1R, CD20 × hL243γ1, HER3 × cMET	MS-TOF, SEC (73%)	HEK293/protein A/0.004–0.03 g/L	Prevent homodimer formation	(49, 52, 53)
Biochemical optimization	F241R, F243S, F241S, F243R (CH2) and C226S, C229S (hinge)	NA	MS-TOF (90%)	*E. coli*/protein A	Avoid covalent bonding of heterodimers	(50)
Electrostatic optimization	K409D (CH3A), D399R (CH3B), K409E (CH3A), D399K (CH3B), and K409E (CH3A), D399R (CH3B)	CD3 × TARTK	LC/MS (98%)	HEK293/Select Sure column and SEC	Prevent homodimer formation	(59)
Electrostatic optimization	K409W, K360E, K370E (CH3A) and D399V, F405T, Q347R, E357N, S364B (CH3B)	VEGFR-2 × MET	SEC (80–90%)	HEK293/protein A	Prevent homodimer formation	(60, 61)
Electrostatic optimization	Q39K, Q105K (VH), S183D (CH1), Q38D (VL), S176D (CL), K392D, K409D (CH3A), and E356K, D399K (CH3B)	HER2 × EGFR	SEC (100%)	Stable CHO/protein A/0.2–0.3 g/L	Cognate chain pairing	(57)
Electrostatic optimization	T350V, L351Y, F405A, Y407V (CH3A) and T350V, T366L, K393L, T394W (CH3B)	HER2 × ErbB2	SEC (95%)	CHO/protein A/0.25 g/L	Improved biophysical properties	(62)
κλ-body	NA	CD19 × CD47, CD47 × EpCAM	Isoelectric focusing/41.5%	PEAK cells/protein A, CaptureSelect immunoglobulin gamma (IgG)-CH1, KappaSelect, and LambdaFabSelect affinity chromatography/1.5 g/L	Exploits variable light chains for generating bispecifics	(63)
Bispecific T-cell engagers	NA	CD3 × 17-1A, CD3 × CD19	Cytofluorometry, ELISA, and SDS-PAGE	CHO/nickel-nitrilotriacetic acid (Ni-NTA) affinity chromatography	Recruitment of T-cells *via* CD3 ligation	(64, 65)
Diabody	NA	HEL × phOx	FPLC	*E. coli*/affinity chromatography/0.3–1.0 mg/L	Easy construction and expression in bacteria	(64–66)
Tandem diabody	NA	CD16 × CD30, CD3 × CD19	SEC	*E. coli*/immobilized-metal chelating chromatography (IMAC)/0.48–0.6 mg/L	Increased valence, stability and activity	(66–68)
Dual-affinity-retargeting	NA	CD16 × CD32B	SEC	HEK293, CHO-S/affinity chromatography	Increased valence and affinity	(67–69)
Bispecific/trispecific killer cell engager	NA	CD16xCD19, CD16xCD19xCD22	SEC	*E. coli*/ion-exchange chromatography	Natural killer cell activation	(69–72)

*CH3A and CH3B mean that these mutations are located on the partner chain*.

*Abbreviations: SLD, scanning laser densitometry; SEC, size exclusion chromatography; MS-TOF, time-of-flight mass spectrometry; FPLC, fast protein liquid chromatography; LCMS, liquid chromatography–mass spectrometry; ESI-MS, electrospray ionization mass spectrometry; DEAE, diethylaminoethanol ion-exchange resin; NA, information not available*.

**Table 2:** **Bispecific antibody candidates under clinical evaluation**.

	Format	Strategy	Target	Clinical development phase	Disease	Company
**bsAb/bsAb Fragment**						
Catumaxomab	Triomab	Quadroma	EpCAM × CD3	Approved by European Medicines Agency Phase-2 (NCT00189345) Phase-2 (NCT01504256) Phase-2 (NCT01246440)	EpCAM^+^ tumor; malignant ascites Platinum refractory epithelial ovarian carcinoma Gastric adenocarcinoma Ovarian cancer	Neovii Biotech AGO Study Group AIO-Studien-gGmbH Grupo Espanol de Investigacion en Cancer de Ovario
Ertumaxomab	Triomab	Quadroma	HER2 × CD3	Phase-1/2 (NCT01569412) Phase-2 (NCT00351858) Phase-2 (NCT00452140) Phase-2 (NCT00522457)	Her2/Neu^+^ advanced solid tumor Advanced metastatic breast cancer	Krankenhaus Nordwest Neovii Biotech
FBTA05	Triomab	Quadroma	CD20 × CD3	Phase-1/2 (NCT01138579)	CLL	Technische Universitat Munchen
RO695688	Crossmab	Knobs-in-holes (KiH)	CEA × CD3	Phase-1 (NCT02324257)	Advanced metastatic CEA^+^ solid tumors	Hoffmann-La Roche
RO5520985	Crossmab	KiH	Ang2 × VEGFA	Phase-1 (NCT01688206)	Advanced or metastatic solid tumors	Hoffmann-LA Roche
RO5520985	Crossmab	KiH	Ang2 × VEGFA	Phase-2 (NCT01688206) Phase-2 (NCT02484690)	Advanced or metastatic solid tumors AMD	Hoffmann-La Roche Hoffmann-La Roche
RG7813	scFv-IgG	NA	CEA × IL-2	Phase-1 (NCT02004106)	Advanced metastatic CEA^+^ solid tumors	
MM-141	scFv-IgG	NA	IGF × HER3	Phase-1 (NCT01733004) Phase-2 (NCT02399137)	Advanced solid tumor Metastatic pancreatic cancer	Merrimack Pharmaceuticals Merrimack Pharmaceuticals
MOR209/ES414	scFv-IgG	NA	PSMA × CD3	Phase-1 (NCT02262910)	Metastatic prostate cancer	Aptevo Therapeutics
LY3164530	Ortho-Fab IgG	Structural optimization	MET × EGFR	Phase-1 (NCT02221882)	Metastatic neoplasm	Eli Lilly and Company
ALX-0061	Nanobody	NA	IL-6R × HSA	Phase-2 (NCT01284569)	Rheumatoid arthritis	Ablynx
ATN-103	Nanobody	NA	TNF × HSA	Phase-2 (NCT01063803)	Rheumatoid arthritis	Ablynx
Blinatumomab	Bispecific T-cell engager BiTE	NA	CD3 × CD19	Approved by Food and Drug Administration Phase-1 (NCT00274742) Phase-2 (NCT01207388) Phase-2 (NCT01209286) Phase-1 (NCT02568553) Phase-2 (NCT02143414) Phase-3 (NCT02003222)	ALL Relapsed NHL Residual ALL Relapsed/refractory ALL Relapsed NHL ALL BCR-ABL^−/−^ B-cell lineage ALL	Amgen GmbH Amgen GmbH Amgen GmbH Amgen GmbH National Cancer Institute National Cancer Institute National Cancer Institute
Solitomab	BiTE	NA	CD3 × EpCAM	Phase-1 (NCT00635596)	Advanced solid tumors	Amgen GmbH
AMG330	BiTE	NA	CD33 × CD3	Phase-1 (NCT02520427)	AML	Amgen GmbH
MT112 (BAY2010112)	BiTE	NA	PSMA × CD3	Phase-1 (NCT01723475)	Castration resistant prostate cancer	Bayer
MT111 (MEDI-565)	BiTE	NA	CEA × CD3	Phase-1 (NCT01284231)	Advanced gastrointestinal adenocarcinoma	Medimmune LLC
AFM11	Tandem diabody (Tandab)	NA	CD19 × CD3	Phase-1 (NCT02106091)	CD19^+^ B-cell NHL	Affimed GmbH
AFM13	Tandab	NA	CD30 × CD16A	Phase-2 (NCT02321592)	Relapsed/refractory Hodgkin’s lymphoma	Affimed GmbH
rM28	Tandab	NA	CD28 × MAPG	Phase-2 (NCT00204594)	Metastatic melanoma	University Hospital, Tuebingen
MGD006	Dual-affinity-retargeting (DART)	NA	CD3 × CD123	Phase-1 (NCT02152956)	Relapsed/refractory AML	MacroGenics
MGD007	DART	NA	CD3 × gpA33	Phase-1 (NCT02248805)	Metastatic colorectal carcinoma	MacroGenics
MGD010	DART	NA	CD32B × CD79B	Phase-1 (NCT02376036)	Healthy subjects	MacroGenics

*Abbreviations: CLL, chronic lymphocytic leukemia; AMD, age-related macular degeneration; CEA, carcinoembryonic antigen; ALL, acute lymphocytic leukemia; NHL, non-Hodgkin’s lymphoma; AML, acute myeloid leukemia; NA, information not available*.

*Source: https://clinicaltrials.gov/ct2/home*.

## Structural Optimization

The KiH (Figure 1B) concept was first proposed by Ridgway et al. (29) to develop Fc heterodimers. It allows the generation of complementary interacting interfaces by manipulating key amino acid residues that participate in the Fc dimeric interaction. Amino acids with small side chain are replaced by ones with larger side chains, thereby creating a knob or protrusion in one chain and *vice versa* to create a hole or socket in the partner chain. Traditionally, a T366Y mutation in one CH3 domain has been used to create a knob while an Y407T mutation in the other CH3 domain (hereafter denoted as Y407′T) gives rise to a hole (29). Such mutations establish intermolecular interactions and promote the heterodimer formation due to knob/hole pairing and bring about the association of two different Fab regions to give rise to a monovalent bsIg. It was also found that complementing existing knob/hole mutations with F405A and T394′W on the knob and hole side, respectively, yielded 92% molecules as heterodimers (29). In an effort to further improve and stabilize the KiH model, a phage-displayed library of CH3 (T366W) knob domain and CH3 (randomized at 366′, 368′ and 407′) hole domain was created by fusing FLAG-tagged CH3-hole to the N-terminal region of the gene-III minor coat protein of M13 bacteriophage (27). The heterodimers were selected by an anti-FLAG antibody and were found to be completely diverse from parent T366W–Y407′A clone. Phage display optimized variants existed preferably as heterodimers and exhibited greater stabilities (27).

The KiH concept suffered from mispairing of heavy and light chains in the heterodimers. Merchant et al. (28) suggested using identical light chains for both Fab domains, which can be further stabilized by engineered disulfide bonds (Figure 1C). They employed a scFv phage-displayed library with restricted light chain usage to identify antibodies recognizing c-MPL and HER3 using common light chains and developed a bispecific c-MPL × HER3 antibody with approximately 95% heterodimer recovery (28).

However, it is not always possible to use a common light chain for developing bispecific molecules because antigen recognition can critically rely on the partner light chain (73, 74). To circumvent the existing light chain mispairing in bsIgs, a methodology termed as “CrossMab” (47) was developed and combined with the KiH technology. In principle, “CrossMab” is achieved by the exchange between heavy and light chains of the Fab portion of one partner in order to generate formats of “CrossMab Fab” (Figure 1D), “CrossMab VH–VL” (Figure 1E), or “CrossMab CH1–CL” (Figure 1F) (47). Such bispecific formats clearly reduced the heavy–light chain mispairing, allowed simultaneous recognition of the two antigens (VEGF-A and Ang2), retained the affinity and stability profiles of the parent antibodies and exhibited antiangiogenic and antitumor activity *in vivo* (47).

Similarly, trispecific antibodies “TriMabs” (Figure 1G) were developed and combined with the KiH technology using N-terminal single-chain Fab (scFab) and C-terminal scFv fusions of Fc region, which avoids the light chain mispairing. The concept was demonstrated by using four specificities (EGFR, IGFR, cMET, and HER3) incorporated into three structural formats (19). TriMabs 1 and 2 engage their respective antigen in a monovalent manner while TriMab 3 binds HER3 in a bivalent fashion. These molecules retained their ability to bind individual antigens and shared kinetic properties with their parent molecules. Besides, the TriMabs were also shown to simultaneously recognize their respective antigens when immobilized on a chip or expressed on human pancreatic adenocarcinoma cells (BXPC-3) and inhibited receptor signaling and tumor growth (19).

An scFab heterodimeric bsIg format (OAscFab-IgG) (Figure 1H) with anti-IGF1R and anti-EGFR specificities has also been described to prevent faulty chain pairing (48). The light chains in the scFab format were attached to the N-terminus of the heavy chain through a 32-residue linker, and heterodimer formation was achieved by KiH mutations. This strategy allowed the recovery of 99% pure heterodimers, which exhibited comparative antigen-binding affinity to the parent antibodies (48).

Further, a conditional functionality was engineered in a bispecific molecule which relied on proteolytic cleavage to generate functional binders. The VH and VL domains incorporating C44 or C100 mutations, respectively, were attached to the C-termini of the Fc region in knob and hole mutant, respectively, *via* a flexible linker. The VH C44 and VL C100 generate a disulfide stabilized Fv (Figure 1I), bypassing the requirement of a VH–VL linker to promote heterodimer formation in such an arrangement (49). Here, the C-terminal attachment does not affect off-rate and instead relieves steric hindrance after proteolytic cleavage. This methodology can be applied to express toxic products in an inactive state in a cell which can be activated later during the processing steps by proteolytic cleavage (49). This design incorporated a proteolytic motif for Pre-Scission within the peptide linker on one of the partner chains. The recognition motif for other proteolytic systems like furin, matrix metalloproteinase-2/9, or urinary plasminogen activator can also be engineered for varying applications (49).

Next, a structure-guided approach was used to generate bsIg by separately expressing monomeric IgG (harboring KiH mutations) in *Escherichia coli*. Equimolar amounts of two monomeric IgGs, when mixed together at a basic pH, resulted mostly in heterodimeric IgGs (50). These constructs also incorporated mutations in CH2 domain residues (F241R/F243S or F241S/F243R) which remain solvent exposed in aglycosylated IgG molecules and the hinge region (C226S/C229S) to avoid covalent association of knob/knob or hole/hole monomers (50).

Recently, Xu et al. (51) utilized a cell-free expression system to generate bsIg based on KiH format. They used anti-CD3, anti-EpCAM, and anti-HER2 antibodies to generate eight bsIgs in four different KiH scaffolds. Among these scaffolds, scFv-KiH and scFv-KiH^r^ (reverse) exhibited superior yields. Moreover, this system allowed optimal heterodimer expression by using equimolar plasmid ratios with an insignificant amount of free chains (51). The Fc fragments with a hole were found to be more stable than Fc-knob, and it was suggested to fuse difficult-to-express proteins to Fc-hole for high-level of expression (51).

Using the KiH platform, Mazor et al. (52) introduced two cysteine pairs in the CH1–CL interface of one of the Fab arm to create disulfide bonds for correct light chain pairing. Almost 100% monovalent bsIg was recovered from variant with CH1 (F126C) and CL (S121C) mutations. These mutations were applied to construct EGFR × HER2 and CD4 × CD70 “DuetMabs” (Figure 1J) (52) and were shown to be devoid of any free chains or fragments and had a molecular mass similar to native IgG. The thermal stabilities of EGFR × HER2 (*T*_m_ = 55°C) and CD4 × CD70 (*T*_m_ = 58°C) antibodies were comparable to the parental antibodies. DuetMabs could simultaneously engage their targets and as a result CD4 × CD70 DuetMab preferentially recognized CD4^+^CD70^+^ T cells over CD4^+^CD70^−^ or CD4^−^CD70^+^ T cells. Similarly, the EGFR × HER2 molecule shared binding kinetics with parental antibodies and also exhibited normal binding to FcγRs, C1q, and FcRn (52).

Based on multistate designs involving molecular modeling, X-ray crystallography validation, and rounds of iteration, Lewis and coworkers (75) identified several favorable mutants across the interface of VH–VL and CH1–CL for formation of an orthogonal Fab interface. In combination with previously reported Fc mutations in favor of the Fc heterodimerization (59), five bsIgs were correctly assembled from six parental mAbs with an average of 93% success rate (75). More recently, Leaver-Fay and coworkers (76) furthered the multistate design strategy by construction of a negative state pool across the CH3 interface *via* protein docking and sequence design. Several novel mutants were discovered to favor the formation of Fc heterodimerization with a purity of more than 90% (76). In combination with the orthogonal Fab interface, four fully bsIgs could be correctly formed with an average success rate of more than 93% in one-step process (76).

CH1–CK heterodimeric scaffold can be used to build bispecific molecules (77, 78). However, it is known that the CH1–CK pair alone is not strong enough to form a stable heterodimer and CH1–CK pair requires cooperation from VH–VL pair for a stable heterodimer assembly (79, 80). To improve the CH1–CK heterodimerization in the absence of VH–VL pair cooperation, Chen et al. employed structure-based design in combination with phage display directed evolution (81). They identified that a S66V mutation in the CH1 domain together with a S69L in the CK domain can stabilize CH1–CK heterodimerization and increase the *in vivo* serum half-life of a previously described CD4-antibody fusion protein (4Dm2m), which targets the CD4-induced (CD4i) coreceptor binding site of the human immunodeficiency virus (HIV) 1 envelope glycoprotein 120 (81–84). It seems that this CH1–CK stabilization may increase the overall stability of 4Dm2m and thus improve its pharmacokinetics. Moreover, the strengthened CH1–CK heterodimerization may pave the way for construction of bispecific molecules based on this scaffold (81).

## Biochemical Optimization

A structure and sequence guided approach identified low energy amino acid pairs in the CH3 domain, which could promote heterodimer formation (54). These mutations were engineered into the design of a new library, which increased the heterodimer yield up to 89%. The strategy was applied to either Fc/single-chain Fv-Fc fusion (scFv-Fc) or scFv-Fc formats utilizing two different CH3 domains favoring heterodimer formation to generate CD16 × HER2 bispecific, which exhibited improved antitumor attributes against the breast cancer cell line SKBr3 in the presence of CD16^+^ natural killer (NK) cells from human peripheral blood mononuclear cells (PBMCs) (54).

In another approach, Strop et al. (55) developed bispecific IgG1 and IgG2 antibodies by oxidation/reduction methodology for chain pairing. They described bispecific mutations (K409 and L368) in the CH3 domain, which allowed the development of bsIg either by coexpressing monomers bearing common light chains or by mixing the purified monomers under mild reducing conditions. Highest bispecificity was achieved by complementing K409/L368 with either IgG1-hinge or IgG2-hinge mutations (55). A CD3 × CD20 bsIg so generated showed *in vitro* cytotoxicity against mouse B-cell lymphoma in the presence of freshly isolated mouse primary T cells. Further, the bsIg mediated a dose-dependent lysis of target cells and also depleted CD20^+^ B cells *in vivo* by T-cell engagement (55).

A similar concept to generate IgG1 bispecific molecules was applied by Labrijn et al. (56). They incorporated a P228S hinge mutation which makes bonds more susceptible to cleavage under reducing conditions and additional mutations in separate human IgG1-CH3 domains to promote Fab-arm exchange. Two mutated antibodies in IgG1 format (anti-EGFR and anti-CD20) were expressed separately and then mixed together in the presence of reducing agent, which resulted in 96% efficient Fab-arm exchange (Figure 1K) (56). The bispecific molecules so generated were stable at 5°C for over a period of 6 months and had comparable pharmacokinetics to their parent antibodies. A dual targeting CD3 × HER2 bispecific IgG1 was also constructed and showed a great enhancement in the T cell-mediated cytotoxicity against breast adenocarcinoma cells (AU565) in comparison to control antibody and *in vivo* tumor growth inhibition in an adoptive transfer xenograft model of gastric carcinoma with freshly prepared human PBMCs (56).

## Electrostatic Optimization

Gunasekaran et al. (59) exploited charged pair based attraction/repulsion in different Fc chains in the scFv-Fc format (Figure 1L). They identified conserved oppositely charged residues in the CH3–CH3 domain interface (D356–K439′, E357–K370′, K392–D399′, and D399–K409′). Among these, the D399–K409′ pair is buried and contributes to CH3–CH3 interaction. Mutations in these key residues (K409D–D399′K, K409D–D399′R, K409E–D399′K, and K409E–D399′R) favored greater than 90% heterodimer formation (59). The strategy was applied to generate a CD3 × TARTK bsAb and shown to kill U87-TARTK^+^ human glioma cell line by CD3 ligation in the presence of human PBMCs (59).

Similarly, Choi et al. (60) substituted conserved and charged amino acids from the core of the CH3 domain with hydrophobic residues to perturb the structural symmetry and designed long-range electrostatic attraction at the edge of the CH3 domain to promote heterodimer formation. A “W–VT” CH3 mutant pair (K409W and D399′V/F405′T) and “ER” CH3 mutant pair (K360E and Q347′R) accounted for 77 and 53% heterodimer species, respectively. This was attributed to the disruption of electrostatic and hydrophobic interactions and increased steric hindrance due to the K409W–F405′T mutations (60). Further, the combination of W–VT and ER mutant increased heterodimer formation up to 91%, enhanced binding to FcRn and imparted parent-like thermal stability. The “ERW–VT” mutant was used to generate MET × VEGFR-2 bispecific scFv (bscFv)-Fc, which bound more strongly to HUVEC cells and inhibited cell growth and VEGF/HGF-mediated ERK/AKT signaling. The MET × VEGFR-2 bsAb induced a twofold greater reduction in tumor volume in MKN45 human gastric cancer xenograft models compared to parent antibodies (60).

Subsequently, Fc heterodimer mutants were isolated based on displayed (CH3A) or secreted (CH3B) yeast libraries from either W–VT mutant or wild-type Fc incorporating K370–E357′/S364′ or D399–K392′/K409′ interaction pairs. The haploid yeast cells carrying individual libraries upon mating resulted in a diploid cell harboring both Fc variants, which if heterodimerized were displayed on the cell surface (61). In addition, these Fc variants carried C228S/C231S mutations to prevent homodimer formation and N297Q to avoid hypermannosylation in yeast (61).

Further, structure-guided design assisted with the computational algorithm and optimized energy function in between the partner chains were used to improve biophysical properties of bsIg molecules. Identified mutations [ZW1—Chain A (T350V/L351Y/F405A/Y407V) and Chain B (T350V/T366L/K393L/T394W)], which favor pure heterodimer formation (95%) and stability comparable to the wild-type Fc, were applied to create bispecific molecules (62). These mutations greatly improved the stability of the heterodimer when exposed to heat, acid, base, agitation, oxidation, freeze-thaw, and varying pH (62).

Electrostatic interactions were also optimized for the correct pairing of the heavy chain and its cognate light chain when coexpressed in the same cell. To achieve this, structure-guided mutations were made by replacing polar or hydrophobic residues of the CH1–CL and VH–VL interfaces with charged amino acids. These mutations promoted correct pairing of the heavy and light chains due to the maximization of electrostatic interactions over H-bonding and Van der Waals forces. The HER2 × EGFR bsIg induced a greater receptor internalization than achieved by the combination of parental antibodies and, furthermore, exhibited enhanced capability to inhibit BXPC-3, PANC-1, and Calu-3 tumors in xenograft models (57).

## Conceptual Advances

Davis et al. (85) exploited the sequence diversity in CH3 domains of IgG and IgA (~47% diverse in humans) and proposed that if diverse patches of IgG and IgA CH3 domains are mutually replaced then they can be heterodimerized (85). This strategy resulted in a unique complementarity among the CH3–CH3 interface. It was experimentally shown by constructing a strand-exchange engineered domain body (SEEDbody) fusion protein [IgG1-hinge-CH2-(SEED-IgA-CH3)], which preferentially associated into heterodimers (85–95%) (85). Depending on the patched sequence, these molecules were referred to as either AG or GA SEED. Further, novel SEEDbodies (Figure 1M) retained normal FcRn and protein A binding due to a specific motif introduced from IgG into the IgA. The major advantage of SEEDbody is to engineer additional specificity *via* scFv fused to the N-terminus of the SEEDbody, which overcomes the faulty light chain pairing (85).

Antibody scaffolds have also been used to develop bispecific pharmacophore fusions “COVX-Bodies” by chemical optimization of bsAbs (86). The principle involved here is to chemically link two pharmacophore peptides *via* branched azetidinone linker followed by an irreversible site-specific covalent fusion to the scaffold antibody. The azetidinone linker interacts with the K94 of Ig heavy chain and establishes an amide bond with the antibody. For example, a humanized IgG1κ aldolase antibody has been used as a scaffold for monovalent display of anti-VEGF and anti-Ang2 peptide pharmacophores to develop a monovalent bispecific COVX-241 (86). The bispecific COVX-241 inhibited VEGF–VEGFR2/Ang2–Tie2 interactions and further exhibited an improved efficacy against colon adenocarcinoma xenograft model as compared to the monospecific COVX-body (86).

Another methodology to develop bsAbs was proposed by Wranik et al. (87), which employed a common light chain and leucine zipper (LUZ-Y) (Figure 1N). They demonstrated heterodimer assembly by creating a point mutation and the addition of a leucine zipper at the heavy chain C-terminus (87). Further, K222A substitution was made in the hinge region to avoid Lys-C endopeptidase-mediated cleavage if used for removing leucine zipper through C-terminal lysine of the CH3 domain. The light chain (VL–CL) was fused to the N-terminus of the heavy chain construct by a non-cleavable linker. However, the leucine zipper did not completely prevent homodimer formation, as an outcome of disproportionate expression in CHO cells most probably due to an incorrect DNA ratio during the transfection (87). Two armed LUZ-Y bispecific molecules hFcεRIα × hFcγRIIb showed desirable binding to respective targets and inhibited histamine release from hFcεRIα/hFcγRIIb^+^ rat basophil leukemia cells when activated by IgE. Similarly, EGFR × HER3 bispecific LUZ-Y inhibited the growth of EGFR/HER3-dependent FaDu cells (87).

Bispecific IgG has also been assembled from a bacterial expression system. Half-IgG (one heavy and one light chain) molecule representing MET (hole mutant) and EGFR (knob mutant) specificities were expressed in bacterial cultures separately. The half-IgGs were purified by protein A affinity chromatography and analyzed to quantify monomeric form by size exclusion chromatography. Two half-IgGs were mixed in the presence of a reducing agent and subsequently oxidized to generate bsAb, which binds to the targets in a monovalent fashion (88). Similarly, a bacterial coculture of two strains expressing individual half-IgGs was shown to be the most rapid and efficient way to produce bsAbs (88). These bsAb molecules also inhibited MET × EGFR driven tumor growth (88).

In another effort, broadly neutralizing anti-HA1 (Ab-002) and HA2 (Ab-005) antibodies against influenza virus A were covalently linked by their C-termini. This methodology involved the fusion of a bacterial sortase recognition motif (LPETGG) *via* G_4_S linker to the C-terminus of the IgG heavy chain. Click chemistry in the form of either cyclooctyne (DIBAC) or azide was used in the presence of sortase to promote covalent association of the two antibody molecules (89). Such covalently linked antibodies remained stable for up to 3 weeks at 37°C and also retained the Fc effector functions. These bispecific covalently linked IgGs contributed to an increased breadth of HA binding and antiviral potency as compared to the monospecific molecules (89).

Further, the light chain pairing was also used to generate bsIg molecules (κλ-bodies) (Figure 1O). This was achieved by coexpressing a single heavy chain with two light chains (κ and λ) through a single expression vector (63). It was presumed that 50% yield would be of κλ heterodimers. The strategy involved a three-step purification to separate (a) whole IgG fraction by protein A/CH1 resin, (b) κ light chain by kappa select resin, and (c) λ light chain by lambda select resin. Fed-batch culture yielded 1.5 g/L of total IgG with 41% being κλ heterodimer (63).

Smith et al. (58) used antibody isotype local chimeras to develop a bsAb that could trigger T cell-mediated B-cell killing. The rationale behind this approach was to exploit the differential protein A binding ability of antibody isotypes. It is known that IgG3 does not bind protein A and harbors a dipeptide Arg-Phe at the CH3 domain that corresponds to IgG1 H435 and Y436, respectively, while IgG1 can bind protein A with high affinity. Moreover, crystal structure of IgG1 in complex with protein A confirms that H435 is involved in interaction with protein A (90). Fc substitutions (H435R, Y436F) were created in anti-CD3 antibody to develop CD3 × CD20 bsAb using a common light chain. Such isotype local chimeras exhibited asymmetric binding to protein A, which was exploited for separation of heterodimers by a pH gradient (58). The yield of the bispecific CD3 × CD20 IgG1 (REGN2280) was 43%. The IgG4 isotype of CD3 × CD20 (REGN1979) exhibited T cell-mediated cytotoxicity against CD20^+^ Raji lymphoma and inhibited the growth of Raji lymphoma cells in NOD SCIDγ (NSG) mice when injected along with the human PBMCs (58).

## bsAb Fragments for T Cell and NK Cell Activation

Besides the different formats of full-length bsAb design, significant effort has also been invested on bsAb fragment designs that lack Fc region or only contain some constant domains. The building blocks of bsAb fragments include nanobodies, human single-domain antibodies, scFvs, and Fabs (18). More than 25 bsAb fragment formats have been documented, and most formats currently under clinical trial for treatment of different tumors involve bridging immune effector T cell (CD3 as the antigen) toward tumor specific antigens (18).

Blinatumomab, the first bsAb fragment and second bispecific molecule approved for therapy, is a bispecific T-cell engager (BiTE) molecule (Figure 1P; Table 2 for clinical status) that is built from two scFvs linked in tandem with a short peptide linker (64, 91). Blinatumomab uses one arm to recognize CD19, which is highly expressed on B-cell acute lymphocytic leukemia (ALL), and the other arm to recruit CD3, which is expressed on T cells, and induces a T-cell–tumor cell contact and potent lysis of tumor cell (64, 91, 92). Interestingly, the BiTE format of CD3 × CD19 bispecific antibody is superior to other formats including diabody (66) (Figure 1Q), tandem diabody (Tandab) (67, 68) (Figure 1R) and quadroma in terms of T cell-mediated tumor cell lysis (93). This finding highlights that the relative orientation and distance between the two scFvs may have significant impacts on how to bridge T cell and tumor cell into close contact so as to trigger T cell-mediated tumor cell lysis. Currently, several bispecific antibody fragments based on BiTE, Tandab, and dual-affinity-retargeting (Figure 1S) (69) formats are under clinical trials involving CD3 and tumor related antigens (18, 94) (Table 2).

Effects of bsAb fragments capable of interacting with NK cells via CD16 have also been investigated. Gleason and coworkers demonstrated the enhanced efficacy of bscFv CD16 × CD19 and trispecific scFv CD16 × CD19 × CD22 constructs in targeting tumor cells by coengaging NK cell effector function (70). These molecules were termed as bispecific and trispecific killer cell engagers (BiKEs and TriKEs) (Figures 1T,U), respectively. Such molecules are constructed by linking specific binders together through a short linker derived from human muscle aldolase. Further, a BiKE construct against CD16 and CD33 has been shown to activate CD16^+^ NK cells to lyse CD33^+^ HL60 target cells (71). Recently, a TriKE construct with additional specificity against IL-15 induced enhanced NK cell cytotoxicity, degranulation, and cytokine production against CD33^+^ HL60 cells *via* increased NK cell proliferation and survival (72). Reusch et al. designed a Tandab-based anti-CD16A × CD30 bispecific tetravalent fragment and found that this format is better than normal monoclonal anti-CD30 IgG, optimized monoclonal anti-CD30 IgG for FcγR binding, and diabody (bispecific and bivalent anti-CD16A and anti-CD30) formats in triggering NK cell lysis of Hodgkin lymphoma cells (CD30 as the antigen) (95, 96). This format of anti-CD16A × CD30 bispecific fragment (known as AFM13) is under phase-2 clinical trial (Table 2) for treatment of refractory or relapsed Hodgkin lymphoma patients (97).

## Monomeric Ig Scaffolds

Much work in the last 5 years has focused on engineering the IgG constant domains as scaffolds. The IgG-Fc region and isolated CH domains have shown promising virtues in terms of small size, antigen targeting, effector function, and serum half-life.

Soluble monomeric Fc (mFc) was developed by destabilizing the CH3–CH3 domain interface. A phage-displayed library was constructed with random mutations introduced within seven key residues known to be involved in CH3 domain homodimerization and selected against binding of protein G first to enrich well-folded mutants and then against human FcRn (16). The selected clones were diverse in their CH3 domain, and monomer promoting mutations were identified (16). These variants with each containing six to seven mutations had considerably lower melting temperature but exhibited comparable stabilities and pH-dependent FcRn binding profiles to that of dimeric Fc (16). The experimental conceptualization was proved by mFc67.3–m36VH fusion (Figure 1V) targeting HIV-1 envelope glycoprotein (ENV) and neutralization of viral isolates (16). Based on the identified mFc mutants, the same group (98) optimized mFc to generate mutants with fewer mutations and improved thermostability while maintaining similar pH-dependent FcRn binding as that of wild-type Fc dimer. Interestingly, the new mFc mutants when expressed either in bacteria or mammalian cells all showed selective binding to only FcγRI but not to other receptors including FcγRIIa, FcγRIIb, FcγRIIIa, and C1q (98). This selectivity attractively made the mFc mutant as a potential carrier to treat chronic inflammatory diseases where inflammatory macrophages showed increased expression of FcγRI (98–103). In addition, this selectivity also excluded unwanted cytotoxicities such as ADCC from FcγRIIIa and CDC from C1q (98).

In another strategy to keep Fc in a monomeric form, asparagine-linked glycan structures were engineered into the CH3–CH3 interface of IgG1 Fc lacking the hinge (104). A mutant incorporating N-glycosylation sites (asparagine at positions 364 and 407) yielded a stable, soluble, and mFc scaffold with wild-type-like FcRn binding activity, which suggests that N-glycosylation promotes functional monomeric state and improves the biophysical characteristics (104).

Smaller scaffolds like CH2 domains also possess interesting properties like a properly folded structure, conformational flexibility, lesser propensity to dimerize, and associated FcRn/C1q binding functions, which can have therapeutic benefits (103, 105). A CH2 domain variant (m01) harboring disulfide bond mutations (L12C and K104C) was found to exist solely as a monomer and remained thermally more stable than native counterparts (105). Further, 50% of monomeric CH2 unfolded at a urea concentration of 6.8 M as compared to 4.8 M for the wild-type CH2 (105). The biophysical attributes of the CH2 domain (m01) were further optimized by developing a shorter version where the unstructured random coil at the N-terminus was shortened by seven residues (m01s) (106). This strategy increased the melting temperature of CH2 isolate up to 82.6°C, enhanced stability in serum and mediated stronger binding to soluble FcRn (106). Similarly, a structure-guided design allowed the engineering of an IgG1-CH3 FcRn recognition motif into the CH2 scaffold to impart enhanced pH-dependent FcRn binding, prolongation of serum half-life, and epithelial transcytosis in comparison to parent CH2 isolate (m01s) (107).

Antigen recognition activity was engineered in CH2 scaffolds carrying mutations in the BC and FG loops by selecting against the HIV-1 envelope glycoprotein (gp120)–CD4 complex (15), which yielded CH2 fragments bearing the same BC but different FG loops, suggesting the importance of the latter in such interactions. One such isolate (m1a1) specifically recognized a highly conserved CD4i epitope on the HIV-1 gp120 protein and neutralized HIV-1 isolates in a cell based pseudovirus assay (15). In another approach, a randomly selected VH domain targeting the HIV-1 gp41 protein was grafted into the BC/FG loops of “m01s” without affecting the flanking regions to develop antigen targeting CH2 domains (m2a1) (108). This domain bound to sp62 peptide of HIV-1 envelope membrane-proximal external region, neutralized HIV-1 isolates, and showed pH-dependent FcRn interaction (108).

Initial success with the mFc and CH2 domains prompted the development of a monomeric CH3 (mCH3) scaffold (103, 109). The seven contact residues of the CH3 domain from the previously reported mFc molecules (16) were mutated, and a combination of mutations was found to be required for CH3 monomer to exist (109). These mutations were responsible for stronger intermolecular hydrophobic interactions, which resulted in an intact, folded and thermally stable CH3 monomer (109). The mCH3 scaffold exhibited significant binding to FcRn and protein G (109). Furthermore, mCH3 fused to a VH domain of an HIV-1 targeting antibody (m36.4) showed satisfactory stability, viral neutralization, and pH-dependent FcRn binding (109). A comparative analysis between the dimeric and monomeric scaffolds has demonstrated increased solubility, comparable serum stability, and higher pH-dependent FcRn binding by monomers (103). These properties make antibody scaffolds promising potential therapeutic candidates.

## Fc Antigen-Binding Fragment

The Fc has also been developed as an antigen-binding domain “Fcab” (Figure 1W) by imparting specificity against antigens through yeast surface display (110). The AB and EF loops of IgG1-CH3 domain were randomly mutagenized, cloned into a yeast surface display vector, and selected against HER2/neu extracellular domain (ECD) for two rounds. The selected binders were again randomized in the AB loop and selected on HER2/neu ECD. One of the isolated Fcab molecules (H10-03-6) showed specificity for binding to HER2, retained binding to FcγRI/protein A, and exhibited an *in vivo* pharmacokinetics profile similar to the native human Fc (110). Post-affinity maturation, Fcab exhibited ~10-fold enhanced binding to the antigen as compared to the parental molecule and elicited NK cell-mediated cytotoxicity *in vitro* against the breast cancer cell line Calu-3, but the potency was ~20-fold weaker than that of Herceptin (110).

Introducing antigen-binding ability into the Fc fragment could compromise stability, and therefore, additional disulfide bonds were engineered within the CH3 domain to provide increased stability (111). Further, these mutations did not affect pH-dependent FcRn binding, which suggests the correct folding of the engineered Fc fragments (111). Similarly, immune modulating activity of Fcab was demonstrated using a HER2 targeting Fcab (HAF3–4), bearing CD16a modulating mutations (111). Both mutants showed binding to the recombinant or cell surface expressed HER2 and had an expected CD16a modulating behavior which correlated with the ADCC potency (111). Later, Woisetschlager and coworkers (112) showed the *in vivo* tumor reduction efficacy of anti-HER2 Fcab (H10-03-6) by simultaneous engagement of the HER2/CD16a and the involvement of ADCC (112).

Since antigen–antibody interaction at an acidic pH can negatively affect drug pharmacokinetics, anti-HER2 Fcab (H10-03-6) variants with weaker binding at pH 6.0 were developed (113). The residues in the AB and EF loops were randomized and selected against HER2-ECD alternately at pH 7.4 and 6.0 using yeast display. The isolated binders exhibited lower affinity at an acidic pH and similarly engaged HER2^+^ cells in a pH-dependant manner (113). A year later, Leung et al. (114) developed Fcab that could degrade HER2 and induce apoptosis. The AB and EF loop residues of IgG-Fc were randomly mutagenized and selected *via* yeast display for binding to HER2-ECD. After affinity maturation, the candidate Fcab (FS102) exhibited an affinity for HER2-ECD that was equivalent to that of pertuzumab and trastuzumab and an extended serum half-life comparable to the native Fc (114). Further, EC_50_ values of 1.1 and 3.3 nM were observed against SKBr3 and HCC1954 breast cancer cells, respectively, and a complete tumor regression was shown using HER2^+^ patient-derived colorectal/gastric cancer xenografts (114). This effect correlated with caspase 3/7 activation in SKBr3 cells in a dose-dependent manner indicating the induction of tumor cell apoptosis (114).

## Challenges in Clinical Development of bsAbs and Fragments

Smaller antibody fragments (nanobody, human single-domain Ab, scFv, or Fab) and bsAb fragments offer a number of advantages over full-length IgG, including ability to penetrate tissue, cost effective and facile manufacturing methods, and high yields (23, 115). However, their small size leads to a shorter serum half-life, lesser tissue retention, and rapid clearance from the blood through kidneys. This is true for blinatumomab, which has a serum half-life of around 2 h while the serum half-life of full-length IgG1 is around 2–3 weeks. Thus, patients need a regimen of at least 3-cycle treatment with each cycle consisting of continuous infusion for 4 weeks in a cycle of 6 weeks (116). On the other hand, the fast clearance of small size bsAb fragments may be desirable in imaging and radioimmunotherapy (117, 118).

Specific approaches can be used to increase the longevity of small bsAb fragments in blood and tissue by (1) fusion of the Fc region of IgG molecules or human serum albumin to prolong the serum half-life. These fusions not only increase molecular size of bsAb fragments and therefore protect them from being excreted out of the body but also mediate binding to the FcRn expressed on the endothelial cells to enter IgG serum stabilization pathway (25, 119–121); (2) multimerization of antibody fragments to increase the molecular size for stabilizing concentration in blood and enhancing the valency of antigen binding (120, 122). The multimerization approach, however, runs a risk of imparting heterogeneity to the molecule and can also lead to undesirable effects by crosslinking of the target receptor (120); and (3) linking of a hydrophobic molecule like polyethylene glycol, a clinically proven technology for serum half-life extension (120, 123). Besides, other polymers like polysialic acid, *N*-(2-hydroxypropyl) methacrylamide, and dextran can also provide protection to the small antibody fragments (25, 120).

Currently, there are more than 60 bsAb formats (18). Of note, the success story of blinatumomab in the format of BiTE, but not in other formats including diabody, Tandab, and quadroma indicates that bispecific molecule design has to consider more than one format from the beginning (93). Moreover, expression and purification of these variable formats require tailored procedures based on each design. This may pose a great challenge in developing bispecific molecules. Table 1 summarizes relevant information from available literatures.

## Final Remarks

Considerable progress has been made in the development of bispecific molecules based on different scaffolds in recent years. Much of this understanding has made it possible to enter an era of bispecific clinical development with two such molecules (catumaxomab and blinatumomab) having been approved for clinical use in humans (18). The field of bsAbs has been evolutionary and revolutionary, which is reflected in our ability to obtain 100% pure heterodimers, the complete evasion of heavy and light chain mispairing, fairly standardized production/purification/analytical methods and, importantly, clear ideas of their potential applications.

The full-length mAbs have relatively poor tissue penetration ability. In contrast, smaller antibody fragments like BiTE, BiKE, and TriKE can effectively penetrate tumor tissue and efficiently recruit and activate immune effector cells to lyse tumor cells (23, 70, 93, 115). Besides, the recent success in delivering a bsIg across the blood–brain barrier by targeting transferrin receptor and β-secretase to reduce brain amyloid-β in non-human primate model may open the era of specific antibody brain delivery and treatment of neurodegenerative diseases (124). In another possibility, combining non-antibody small fragments such as lambody, affibody, and aptamer with current antibody scaffolds may expand the treatment arsenal of bispecific molecules.

Moreover, monomeric Ig domain scaffold and Fcab are potent new formats to target difficult to reach sites in the body and to add additional antigen specificity along with enhanced stability, effector function, and extended serum half-life. These properties make them suitable for clinical validation.

In the future, bispecific molecules based on various scaffolds will represent an indispensable class of therapeutic options to treat a variety of clinical indications. However, efforts to improve production and purification on an industrial scale must continue to ensure harvesting of the full benefits of these entities.

## Author Contributions

DW conceived the topic; DW, HL, and AS wrote the manuscript; HL and AS contributed equally in writing the manuscript; DW and SS revised the manuscript.

## Conflict of Interest Statement

The authors declare that the research was conducted in the absence of any commercial or financial relationships that could be construed as a potential conflict of interest.
